# Therapeutic Effects of hiPSC-Derived Glial and Neuronal Progenitor Cells-Conditioned Medium in Experimental Ischemic Stroke in Rats

**DOI:** 10.3390/ijms22094694

**Published:** 2021-04-29

**Authors:** Diana Salikhova, Tatiana Bukharova, Elvira Cherkashova, Daria Namestnikova, Georgy Leonov, Maria Nikitina, Ilya Gubskiy, Gevorg Akopyan, Andrey Elchaninov, Konstantin Midiber, Natalia Bulatenco, Victoria Mokrousova, Andrey Makarov, Konstantin Yarygin, Vladimir Chekhonin, Liudmila Mikhaleva, Timur Fatkhudinov, Dmitry Goldshtein

**Affiliations:** 1 Research Centre for Medical Genetics, 115522 Moscow, Russia; bukharova-rmt@yandex.ru (T.B.); golerus@gmail.com (G.L.); bnv695@gmail.com (N.B.); victoria-mok@yandex.ru (V.M.); dvgoldshtein@gmail.com (D.G.); 2 Research Institute of Human Morphology, 117418 Moscow, Russia; mary.krutikova@gmail.com (M.N.); elchandrey@yandex.ru (A.E.); midiberkonst@gmail.com (K.M.); mikhalevalm@yandex.ru (L.M.); tfat@yandex.ru (T.F.); 3 Department of Neurology, Neurosurgery and Medical Genetics, Pirogov Russian National Research Medical University, 117997 Moscow, Russia; tchere@yandex.ru (E.C.); dadnam89@gmail.com (D.N.); gubskiy.ilya@gmail.com (I.G.); anvitmak@yandex.ru (A.M.); chekhoninnew@yandex.ru (V.C.); 4 Radiology and Clinical Physiology Scientific Research Center, Federal State Budgetary Institution “Federal Center of Brain Research and Neurotechnologies of the Federal Medical Biological Agency”, 117997 Moscow, Russia; gev000rg@mail.ru; 5 Institute of Biomedical Chemistry, 119121 Moscow, Russia; kyarygin@yandex.ru; 6 Russian Medical Academy of Continuous Professional Education, 125993 Moscow, Russia; 7 Department of Histology, Cytology and Embryology, Peoples’ Friendship University of Russia, 117198 Moscow, Russia

**Keywords:** ischemic stroke, MCAO, glial progenitor cells, neuronal progenitor cells, induced pluripotent stem cells (iPSCs), conditioned medium

## Abstract

Transplantation of various types of stem cells as a possible therapy for stroke has been tested for years, and the results are promising. Recent investigations have shown that the administration of the conditioned media obtained after stem cell cultivation can also be effective in the therapy of the central nervous system pathology (hypothesis of their paracrine action). The aim of this study was to evaluate the therapeutic effects of the conditioned medium of hiPSC-derived glial and neuronal progenitor cells in the rat middle cerebral artery occlusion model of the ischemic stroke. Secretory activity of the cultured neuronal and glial progenitor cells was evaluated by proteomic and immunosorbent-based approaches. Therapeutic effects were assessed by overall survival, neurologic deficit and infarct volume dynamics, as well as by the end-point values of the apoptosis- and inflammation-related gene expression levels, the extent of microglia/macrophage infiltration and the numbers of formed blood vessels in the affected area of the brain. As a result, 31% of the protein species discovered in glial progenitor cells-conditioned medium and 45% in neuronal progenitor cells-conditioned medium were cell type specific. The glial progenitor cell-conditioned media showed a higher content of neurotrophins (BDNF, GDNF, CNTF and NGF). We showed that intra-arterial administration of glial progenitor cells-conditioned medium promoted a faster decrease in neurological deficit compared to the control group, reduced microglia/macrophage infiltration, reduced expression of pro-apoptotic gene *Bax* and pro-inflammatory cytokine gene *Tnf*, increased expression of anti-inflammatory cytokine genes (*Il4*, *Il10*, *Il13*) and promoted the formation of blood vessels within the damaged area. None of these effects were exerted by the neuronal progenitor cell-conditioned media. The results indicate pronounced cytoprotective, anti-inflammatory and angiogenic properties of soluble factors secreted by glial progenitor cells.

## 1. Introduction

Deterioration of brain function in ischemic stroke is overwhelming. The acute ischemic damage to brain tissue causes focal and generalized neuronal death with diverse neurological sequelae [1]. The efficacy of treatment and rehabilitation of the patients after acute cerebrovascular episodes remains extremely low. In the absence of etiological treatment in the subacute and chronic phases of the disease, it is important to boost the rehabilitation of patients by stimulating nervous tissue repair [2]. New efficient maintenance therapies for cerebrovascular diseases are in great demand.

Stem cells represent an effective tool of regenerative medicine. The efficiency of cell therapies is based on two fundamental properties of transplanted cells—the ability to replace the destroyed differentiated cells (the replacement mechanism) and the ability to secrete regulatory molecules participating in the processes of inflammation, angiogenesis and regeneration (the paracrine mechanism) [3,4]. The paracrine concept of cell therapies is currently taking the lead [5,6]. Accordingly, the conditioned culture media attract increasing interest as a source of biologically active molecules. Neurotrophins, cytokines and other regulatory molecules accumulated in the cell culture media can exert complex positive effects in various pathological conditions. The great potentiality of stem cell-conditioned media in the treatment of neurodegenerative and vascular diseases has been demonstrated in a number of studies [7,8,9].

Neuronal and glial progenitor cells (respectively, NPCs and GPCs) can be derived from human induced pluripotent stem cells (hiPSCs). Though positive effects of their conditioned medium on the damaged nervous tissue appear quite plausible, they have not been studied yet, and no substantive information on this subject can be found in the literature.

Here, we report the results of comparative assessment of the therapeutic effects of the conditioned medium of hiPSC-derived NPCs and GPCs. The efficacy of the NPC- and GPC-conditioned medium was evaluated in vivo by using the rat middle cerebral artery occlusion (MCAO) model of ischemic stroke.

## 2. Results

### 2.1. Characterization of Obtained Cell Cultures

**Figure 1:**
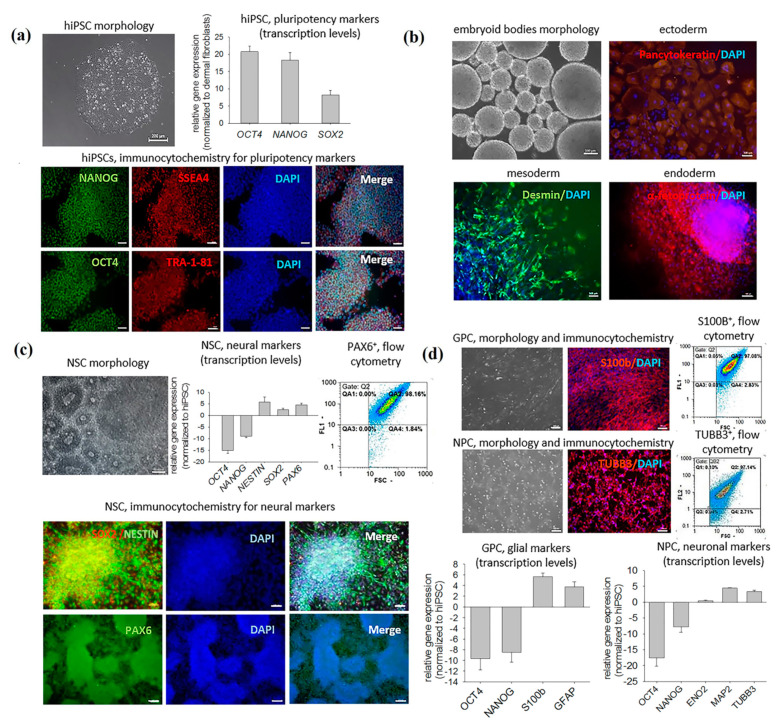
Morphology and characterization of the cultures differentiating towards neuronal and glial phenotypes. (**a**) Characterization of hiPSCs: phase−contrast microscopy, immunocytochemistry for the pluripotency markers (SSEA4, TRA−1−81, OCT4, NANOG), relative gene expression levels for OCT4, SOX2 and NANOG were calculated based on the housekeeping genes GAPDH and ACTB, and then normalized to dermal fibroblasts. (**b**) Functional assay of pluripotency: phase−contrast microscopy of the embryoid bodies and immunocytochemistry of spontaneously differentiated hiPSC derivatives with ectoderm−, mesoderm− and endoderm− specific antibodies (anti−pan−cytokeratin, anti−desmin and anti−α−fetoprotein, respectively). (**c**) Characterization of NSCs: phase−contrast microscopy, immunocytochemistry, flow cytometry for PAX6+ cells and relative gene expression levels for neural markers (PAX6, NESTIN, SOX2), which were calculated based on the housekeeping genes GAPDH and ACTB, and then normalized to hiPSC. (**d**) Characterization of GPCs and NPCs: phase−contrast microscopy, immunocytochemistry for S100B (glial marker) and βIII tubulin (TUBB3, neuronal marker), flow cytometry for S100B+ and TUBB3+ cells and relative gene expression levels for glial and neuronal markers, which were calculated based on the housekeeping genes GAPDH and ACTB, and then normalized to hiPSC. The results are shown for the GPCs and NPCs derived from a single hiPSC line.

The obtained hiPSCs were morphologically similar to human embryonic stem cells; displayed a high nuclear-cytoplasmic ratio; expressed pluripotency marker genes OCT4, NANOG and SOX2; and were immunopositive for OCT4 and NANOG transcription factors and SSEA4 and TRA-1-81 proteoglycans (Figure 1a). The cells also showed the capacity of spontaneous differentiation into the derivatives of all three germ layers (ectoderm, endoderm and mesoderm), confirming their pluripotency at the functional level (Figure 1b). The addition of the appropriate inducers caused differentiation of hiPSCs into neural stem cells (NSCs)—small, densely growing cells prone to the formation of 3D rosette-like structures. The NSC phenotype was confirmed immunocytochemically and by a PCR assay. NSCs expressed molecular markers of neural differentiation (PAX6, SOX2 and NESTIN) and were immunopositive for the corresponding proteins. The differentiation efficiency (evaluated by flow cytometry as the fraction of PAX6-positive cells) constituted 98 ± 1.8% (Figure 1c). Upon stimulation with glial inducers, NSCs acquired spindle-shaped morphology with an uneven outline and large oval nuclei. Stimulation of NSCs with neuronal inducers promoted the outgrowth of neurites up to three cell diameters long. Glial progenitor cells (GPCs), unlike the hiPSCs, expressed S100B and GFAP, and 97 ± 2.8% of them were S100B protein positive. Neuronal progenitor cells (NPCs) had smaller nuclei, unlike the hiPSCs, expressed neuronal marker genes TUBB3, MAP2 and ENO2, and 96 ± 3.4% of them were βIII tubulin positive (Figure 1d).

### 2.2. Comparative Study of GPC- and NPC-Conditioned Medium

**Figure 2:**
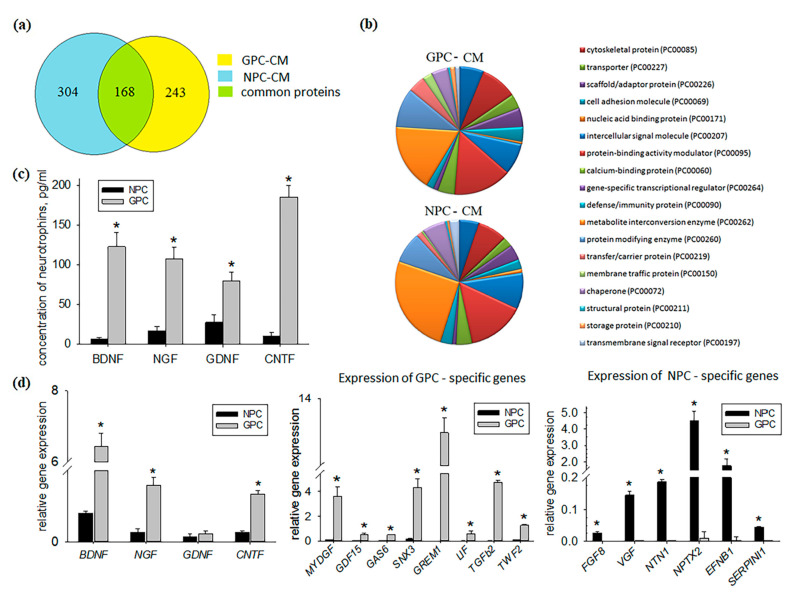
Comparative analysis of the secretory activity and transcription profiles of the hiPSC-derived NPC and GPC cultures. (**a**) Proportions of unique and common proteins in NPC and GPC secretomes. In total, 136 (45%) proteins in NPC-CM and 75 (31%) proteins in GPC-CM were specific. (**b**) PANTHER protein class charts for NPC and GPC secretomes. (**c**) Secretion of neurotrophins by NPC and GPC (ELISA). The highest concentrations of BDNF, NGF, CNTF and GDNF contain GPC-CM. (**d**) Expression levels of NPC and GPC markers (PCR assay). The data are presented as mean ± SD. Asterisks (*) indicate significant differences (*p* ≤ 0.05). These results are shown for the GPCs and NPCs derived from a single hiPSC line.

All secreted proteins by NPCs and GPCs are presented in supplementary Table S1, and cell type-specific biomolecules are presented in Table 1. The NPC-conditioned medium (NPC-CM) contained more protein species than GPC-conditioned medium (GPC-CM). In total, 136 (45%) out of 304 protein species identified in NPC-CM could not be found in GPC-CM (Figure 2a). A considerable number of NPC-specific peptides reportedly possess neuroprotective properties (e.g., tissue inhibitor of metalloproteinases 2, Wnt family member 5A, neuropilin-1, secretogranin-2, platelet-derived growth factor D) [10]. NPC secretomes also comprised apolipoprotein A1, which is known to attenuate the activity of neutrophils [11] and the osteopontin-a chemotactic protein involved in the activation of immune cells and cytokine production [12,13]. Moreover, NPC secreted factors lactadherin and glypican-1 that promote angiogenesis [14,15] (Table 1).

Of the 243 protein species identified in GPC-CM, 75 (31%) were specific to it, i.e., not found in NPC-CM (Figure 2a). The list of the GPC-CM-specific proteins included important regulators of cell survival (growth arrest-specific protein 6, heat shock 70 kDa protein 4, heat shock protein 105 kDa, leukemia inhibitory factor, gremlin 1, Hsc70-interacting protein) [16,17,18]. GPC secretomes also comprised growth and differentiation factor 15 and SH3 domain-binding glutamic acid-rich-like protein 3, which is known to exert pronounced anti-inflammatory effects [19,20]. Proteins involved in angiogenesis were also found such as myeloid-derived growth factor and transforming growth factor-β2 [21,22] (Table 1).

Comparative analysis of GPC- and NPC-CM also revealed differences in the content of certain functional classes of proteins. For instance, the levels of transfer/carrier proteins (PC00219) and membrane traffic proteins (PC00150) in the GPC-CM were, respectively, 2.8 times and 5 times higher than in the NPC-CM (Figure 2b).

Neurotrophins are secretory proteins maintaining the viability of neurons and stimulating their development and activity. In this study, the neurotrophins remained undetected by the proteomic analysis, apparently because of their low concentrations in CM; however, those concentrations were within the sensitivity limit of ELISA. Neurotrophin concentrations in the undiluted GPC-CM and NPC-CM were in the pg/mL range, which is lower than required for therapeutic activity. At the same time, concentrations of BDNF (brain-derived neurotrophic factor), NGF (nerve growth factor), CNTF (ciliary neurotrophic factor) and GDNF(glial cell-derived neurotrophic factor) in the GPC-CM were, respectively, 19-, 12-, 18- and 3 times higher than in the NPC-CM (Figure 2c). It should be noted that neurotrophin genes were expressed by both cultures, and the expression levels of BDNF, CNTF and NGF were higher in GPCs compared to NPCs (Figure 2d). GPCs showed high expression levels of GREM1 (gremlin 1), GAS6 (growth arrest-specific protein 6), LIF (leukemia inhibitory factor), TWF2 (twinfilin 2), SNX3 (sorting nexin 3), MYDGF (myeloid-derived growth factor), TGFB2 (transforming growth factor beta 2), and GDF15 (growth and differentiation factor 15) genes (Figure 2d). In NPCs, expression of these genes was low or undetectable. A number of genes, including FGF8 (fibroblast growth factor 8), NTN1 (netrin 1), NPTX2 (neuronal pentraxin 2), EFBN1 (ephrin B1), SERPINI1 (neuroserpin 1) and VGF (neuro-endocrine specific protein VGF), were expressed specifically by NPC cultures (Figure 2d).

### 2.3. Therapeutic Effects of the Intra-Arterial Infusion of NPC-CM and GPC-CM in the Experimental Ischemic Stroke 

**Figure 3:**
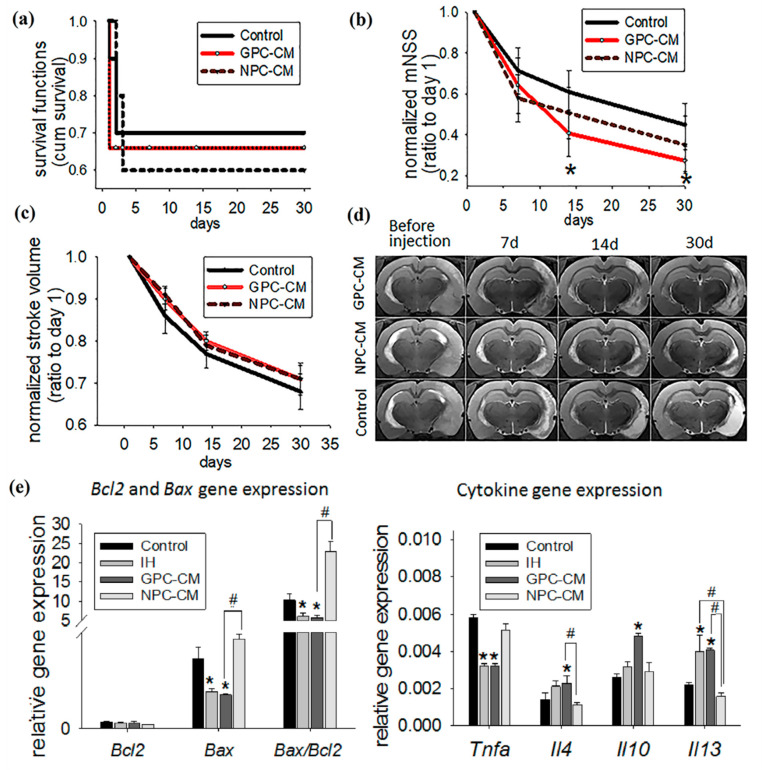
Therapeutic effects of NPC-CM and GPC-CM assessed by the overall survival dynamics, the stroke volume (evaluated by MRI), the dynamics of the neurological deficit (evaluated by mNSS for rodents) and relative gene expression levels of pro- and anti-inflammatory cytokines and apoptosis regulator (*Bax*, *Bcl2*). In this experiment, the following number of animals were used: NPC-CM group (*n* = 12), GPC-CM group (*n* = 12) and the non-conditioned media-treated control (*n* = 10). (**a**) Kaplan–Meier survival curves for three groups. Note that animals in both groups died mostly within the first 3 days after MCAO, presumably because of the formation of vasogenic edema. No significant difference between the two groups was found. (**b**) mNSS dynamics (normalized by day 1 values). IA administration of GPC-CM enhanced functional recovery of the ischemic brain. The graph demonstrates the reduction of the neurological deficit in the GPC-CM group compared to the control group on the 14th and 30th day. (**c**) Infarct volume dynamics (normalized by day 1 values). IA administration of NPC-CM and GPC-CM had no significant effect on the rate of the reduction of the infarct zone volume. (**d**) T2-weighted brain images (T2-WI) for three groups (NPC-CM-treated, GPC-CM-treated and the non-conditioned media-treated control). (**e**) Relative gene expression levels of *Bax*, *Bcl2* and their ratio; pro- and anti-inflammatory cytokines. IA administration of GPC-CM reduced expression of *Bax*, *Tnfa* and increased expression of *Il4*, *Il10* and *Il13* genes. The data are presented as mean ± SD with asterisks (*) indicating significant differences with the control (*p* ≤ 0.05). Hashes (#) indicate significant differences (*p* ≤ 0.05) between GPC-CM- and NPC-CM-treated animals.

Kaplan–Meier survival curves for three groups (NPC-CM-treated, GPC-CM-treated and the non-conditioned media-treated control) are shown in Figure 3a. All deaths occurred within 3 days after the stroke and were associated with vasogenic cerebral edema. CM infusions had no effect on survival.

The neurological deficit was assessed using modified neurological severity scores (mNSS) for rats [23]. MNSS is one of the most reliable and commonly used neurological functional scales that includes motor, sensory, reflex and coordination tests. The score is ranged from 0 to 18 points and can be used to assess the severity of stroke in rodents: the higher the score, the more impaired the rat is. In the present study, mNSS were recorded in dynamics at the following time points: immediately before the infusion (day 1), and on days 7, 14 and 30 post-infusion (p/i). Neurological deficit reached its maximum by 24 h after the acute focal ischemia modeling in all groups; its progression was associated with the cerebral infarct development. After that, the neurological deficit underwent regression in all groups; the highest rates of functional recovery were observed during the initial 2 weeks of the experiment. The scores for the GPC-CM group on days 14 and 30 p/i were, respectively, 1.5-times and 1.6-times lower as compared with the control group; these differences were significant (Figure 3b). By contrast, no significant differences in mNSS between the NPC-CM group and the control group were observed during the experiment. The data indicate enhanced functional recovery of brain function in response to the infusion of GPC-CM during the acute period of ischemic stroke.

The stroke volume was evaluated by MRI, with T2-weighted brain magnetic resonance images acquired at the same time points used for the evaluation of the effects of the CM infusion. The reduction of the infarct volume was pronounced in all groups; no significant differences between the groups were revealed over the 30 day period (Figure 3c,d).

To understand the CM-mediated therapeutic effects at the molecular level, expression of apoptosis- and inflammation-related molecular markers in brain tissues was studied by a PCR-based assay (Figure 3e). The GPC-CM group showed significantly reduced expression of the pro-apoptotic gene *Bax* compared with the control group. By contrast, expression levels of *Bax* in the ischemized tissue of the NPC-CM group were 2.4-times higher as compared with the intact brain tissue in the contralateral cerebral hemisphere (IH) and the GPC-CM group; these differences were also significant. At the same time, no significant differences in *Bcl2* expression were observed between the groups (Figure 3e). The intracellular ratio of *Bax/Bcl2* expression can profoundly influence the ability of a cell to respond to an apoptotic signal. According to this concept, a cell with a high *Bax*/*Bcl2* ratio will be more sensitive to a given apoptotic signal when compared to a similar cell type with a low *Bax*/*Bcl2* ratio [24]. By comparing the expression level of these genes on day 30 p/i, we found that in the GPC-CM and IH groups, there was a decreased *Bax*/*Bc-2* ratio, but not in the NPC-CM group. Infusion of NPC-CM enhanced the *Bax*/*Bcl2* ratio compared with the GPC-CM (Figure 3e).

Vascular necrosis causes secondary damage to brain parenchyma due to the continuous inflammatory reaction accompanied by the elevated expression of the *Tnfa* gene. Infusion of GPC-CM specifically reduced *Tnfa* expression in the affected brain parenchyma; the difference with the control was statistically significant. In addition, expression levels of the anti-inflammatory cytokine genes *Il4, Il10* and *Il13* were significantly elevated in the ischemized brain tissues of GPC-CM-treated animals compared with the control group. By contrast, expression levels of *Tnfa, Il4, Il10* and *Il13* in the ischemized brain tissues of NPC-CM-treated animals were comparable with the control group (Figure 3e).

**Figure 4:**
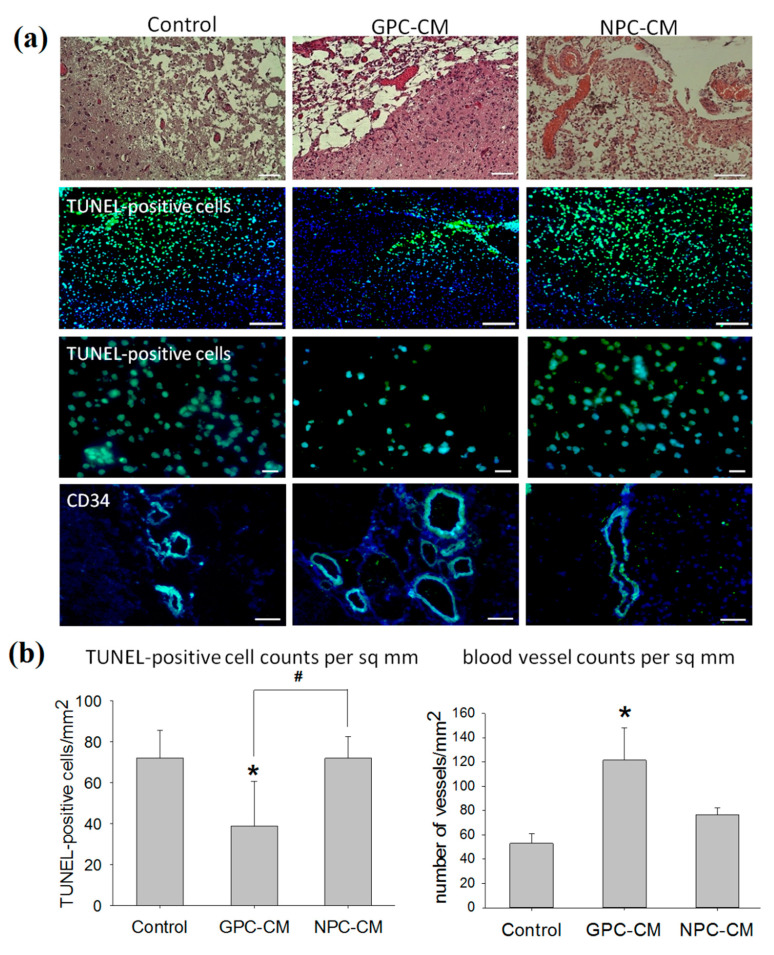
Post-ischemic repair-related effects of the conditioned media. In this experiment, the following number of animals were used: NPC-CM group (*n* = 12), GPC-CM group (*n* = 12) and the non-conditioned media-treated control (*n* = 10). (**a**) Representative routine histology images (H&E), immunohistochemistry with anti-CD34 antibodies and TUNEL-labelled cell death (the nuclei counterstained with DAPI). Scale bar, 200 and 500 µm. (**b**) Counts of blood vessels CD34+ and TUNEL-positive cells per sq mm of the damaged brain tissue section area. The graph demonstrates the increment of number blood vessels and reduction of the number of TUNEL-positive cells in the case of GPC-CM administration. The data are presented as mean ± SD. Asterisks (*) indicate significant differences (*p* ≤ 0.05) with the controls. Hashes (#) indicate significant differences (*p* ≤ 0.05) between GPC-CM- and NPC-CM-treated animals.

To investigate the effects of CMs on apoptosis after stroke, we analyzed apoptotic TUNEL-positive cells. The number of apoptotic cells in the GPC-CM-injected group was lower than that in the control and NPC-CM groups (Figure 4a,b).

As demonstrated by the histological study, the infusion of GPC-CM supported angiogenesis. The counts of formed perfused blood vessels (CD34+ cells) in the ischemized brain tissues of GPC-CM-treated animals were the highest; the difference with the control group was significant (*p* ≤ 0.05, Figure 4a,b).

**Figure 5:**
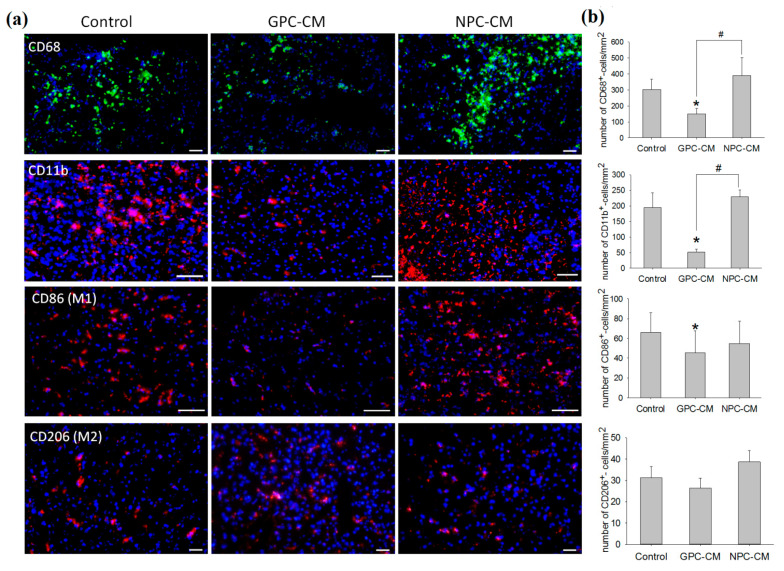
Post-ischemic inflammation-related effects of the conditioned media. Assessment of total M1 and M2 macrophages/microglia. In this experiment, the following number of animals were used: NPC-CM group (*n* = 12), GPC-CM group (*n* = 12) and the non-conditioned media-treated control (*n* = 10). (**a**) Immunohistochemistry with anti-CD68, anti-CD11b, anti-CD86 (M1) and anti-CD20 (M2) antibodies (the nuclei counterstained with DAPI). Scale bar, 100 and 200 µm. (**b**) Counts of macrophages/microglia CD68+, CD11b+, CD86+ and CD206+ cells per sq mm of the brain tissue section area. The graph demonstrates the reduction of the total number of macrophages/microglia (CD68+ and CD11b+ cells) and M1 macrophages in the case of GPC-CM administration. There were no significant differences in the number of M2 macrophages between the groups. The data are presented as mean ± SD. Asterisks (*) indicate significant differences (*p* ≤ 0.05) with the controls. Hashes (#) indicate significant differences (*p* ≤ 0.05) between GPC-CM- and NPC-CM-treated animals.

CD68 is a cell surface glycoprotein highly expressed on phagocytic cells of the resident microglia and infiltrating monocytic macrophages. CD11b is expressed on the surface of cells involved in the innate immune system, including monocytes, macrophages and microglia. An accumulation of CD68+ and CD11b+ cells in the ischemized brain area was detected at the autopsy on day 30 p/i in all groups. However, this effect was significantly alleviated in GPC-CM-treated animals, as the numbers of accumulated CD68+ and CD11b+ cells were lower compared with other groups. No such alleviation was observed in the NPC-CM group where the counts of CD68+ and CD11b+ cells in the damaged area were significantly higher compared with the GPC-CM group (Figure 5a,b).

Single cells immunopositive to the marker of macrophages of the M2 phenotype-CD206 (mannose receptor) were observed on day 30 p/i of the experiment in the damaged area of brain. Moreover, there are no statistically significant differences in the number of CD206+ cells in all of the groups. However, the administration of GPC-CM significantly reduced M1 macrophages (CD86+ cells) compared with the control group, which was not noticed in the GPC-CM infusion (Figure 5a,b).

## 3. Discussion

hiPSC-derived NPCs and GPCs produce and secrete numerous regulatory proteins and peptides. NPC-CM and GPC-CM partially overlap, but large proportions of proteins in their secretomes are highly specific. Although no straightforwardly matching data are available from the literature, the obtained results can be indirectly compared with other studies. For instance, the obtained NPC secretory profiles show 71% overlap with the results of Mendes-Pinheiro et al., who applied a proteomic approach to the primary cultures of human neuronal progenitors and identified 538 secreted proteins [25]. Significant overlaps of the obtained GPC secretory profiles with the corresponding data for primary cultures of human astrocytes should also be noted [26,27,28,29,30,31]. Both NPC and GPC cultures secreted low amounts of neurotrophins (BDNF, CNTF, NGF and GDNF). However, at the mRNA level, GPC cultures showed higher expression of BDNF, CNTF and NGF, which is consistent with higher rates of neurotrophin secretion by GPC cultures reported elsewhere [32,33,34].

Intra-arterial infusion of GPC-CM during the recovery after the experimental ischemic stroke in the rat model was advantageous. Compared with NPC-CM and the non-conditioned media, GPC-CM accelerated functional recovery of the brain: it reduced the neurological deficit and downregulated the expression of pro-apoptotic gene Bax. Besides, we have shown the significant decrease in the ratio of *Bax*/*Bcl-2* expression in the GPC-CM group. These data were confirmed by the data of the TUNEL analysis that demonstrated a reduction in the number of apoptotic cells. Although the GPC-CM administration had no significant effects on the infarct volume dynamics, its anti-inflammatory and pro-angiogenic effects were pronounced. These effects included decreased counts of phagocytic cells (microglia/macrophages), elevated expression levels of anti-inflammatory cytokine genes (*Il4*, *Il10*, *Il13*), a reduced expression level of *Tnfa* and an increased number of formed blood vessels. Despite the fact that we found differences between the GPC-CM and NPC-CM groups in the increased numbers of formed blood vessels and the reduction of phagocytic cell numbers, we did not reveal significant differences in the neurological deficit between these two groups within 30 days. It is likely that these effects may appear later and that a longer observation period is required. Interestingly, the injection of both CM does not influence the number of macrophages M2 phenotype, but administration of GPC-CM reduced the number of macrophages M1 phenotype. We suggested that GPC-CM might have an effect on macrophage cells by changing their phenotype.

Intra-arterial infusion of NPC-CM had no pronounced functional effects. Moreover, expression levels of *Bax*, *Bax/Bcl2* ratio and the counts of CD68+, CD11b+ and TUNEL-positive cells within the affected brain area of the NPC-CM-treated animals were significantly higher than for the GPC-CM-treated group. We did not observe a significant difference between the two groups in *Tnfa* gene expression. However, we noticed a certain tendency of increased expression of this gene in the NPC-CM group, and in the case of a larger number of animals in a future experiment, this difference could probably be identified.

Thus, the obtained results indicate pronounced neuroprotective, anti-inflammatory and pro-angiogenic properties of GPC-CM as compared with NPC-CM. The difference is apparently related to the unique secretory profiles of the GPCs. The obtained results are consistent with previous reports demonstrating the efficiency of stem and progenitor cell-conditioned media in vivo. For instance, administration of NSC-conditioned medium to the rats with an experimental ischemic stroke promoted alleviation of neurological deficits and reduction in the infarct volume compared with the control group [35]. Intravenous infusion of the bone marrow mesenchymal stem cell-conditioned medium to the rats with an experimental ischemic stroke significantly enhanced functional recovery and alleviated the microglial/macrophage infiltration of brain tissue [9]. As demonstrated by Hicks et al., the transplanted primary NSCs actively produce angiopoietin 1 (Ang1), which promotes an increase in the amount of microvessels in the ischemic area of the brain [36]. However, the effects of CM of the hiPSC-derived NPCs and GPCs on the recovery of the brain tissue after an experimental ischemic stroke are reported here for the first time.

According to the results of this pilot study, the hiPSC-derived GPC-CM represent a promising candidate for clinical studies, as it can be obtained in virtually unlimited numbers from the patient’s own cells (e.g., dermal fibroblasts) and used as autologous transplants. However, for the potential translation of this technology to the clinic, it is reasonable to clarify a number of issues. Currently, it has not been established whether there are differences in the therapeutic efficacy of CM derived from different hiPSC lines. Moreover, further research is needed to determine the optimal dose of CM, the time “window” for intra-arterial administration and the cellular/molecular mechanisms that promote the recovery after stroke.

## 4. Materials and Methods

### 4.1. Animals

The animal studies were approved by the local Ethical Committee of the Pirogov Russian National Research Medical University (Protocol number 13/2020 from October 8, 2020) and were carried out in accordance with EU Directive 2010/63/EU. All animal studies were reported according to ARRIVE guidelines. Animals were obtained from AlCondi, Ltd. Moscow, Russia. Adult male Wistar rats of 250–300 g body weight (*n* = 34) were used in the experiments. Males were chosen to avoid the potential neuroprotective effects of estrogen on stroke formation [37]. The rats were housed in plastic cages (4–5 per cage) under a 12/12 h light/dark cycle with maintenance of a temperature of 24–26 °C and humidity of 40–70%. During the experiments, laboratory rats were kept in comfort facilities and were given ad libitum access to drinking water and food (standard rodent chow). All efforts were made to minimize the number of animals and to exclude pain and other unpleasant effects for the animals. All surgical procedures and MRIs were conducted under isoflurane inhalation anesthesia (Aerrane, Baxter HealthCare Corporation, Deerfield, IL, USA) supplied by the animal anesthesia system (E-Z-7000 Classic System, E-Z-Anesthesia Systems, USA): 3.5–4% isoflurane mix with atmospheric air for the induction of anesthesia and 2–2.5% isoflurane/air mix for its maintenance. For euthanasia at the end of the experiment, the animals were placed in an induction chamber (E-Z-7000 Classic System, E-Z-Anesthesia Systems, Palmer, PA, USA) and inhalation anesthesia was performed with a lethal dose of isoflurane. Afterwards, animals were additionally injected with a lethal dose of Zoletil (Virbac, Carros, France).

### 4.2. Experimental Design

The main goal of this study was to assess the primary therapeutic efficacy of GPC-CM or NPC-CM after a stroke in rats. For this, an experimental ischemic stroke was modeled in all male Wistar rats by a temporary 90 min occlusion of the right middle cerebral artery. At 24 h after stroke modeling, the animals (*n* = 34) were randomly attributed to 1 of the following 3 groups: the control group (*n* = 10), which received intra-arterial infusions of the non-conditioned DMEM/F12-based medium; one group of CM recipients (*n* = 12), which received intra-arterial infusions of GPC-CM (50 µg/mL of total protein); and another group of CM recipients (*n* = 12), which received intra-arterial infusions of NPC-CM (50 µg/mL of total protein). The doses of GPC-CM and NPC-CM were chosen according to the literature data [38,39]. It is worth noting that the optimal “time window” for intra-arterial transplantation of stem/progenitor cells or their conditioned medium has not been firmly established yet [40,41]. In the current study, the “time window” was chosen on the basis of several points: (1) the literature data [42,43,44]; (2) the increased permeability of the blood–brain barrier within 6–48 h after stroke onset [45]; (3) the duration of the therapeutic window for clinical reperfusion methods, that does not exceed 24 h [46]; (4) the results of our previous study, where we demonstrated significant therapeutic efficacy after intra-arterial transplantation of neural precursor cells 24 h after MCAO modeling in rats [47]. In the current study, the assessment of the therapeutic effects of GPC-CM or NPC-CM was carried out immediately before the infusion (day 1) and on days 7, 14 and 30 post-infusion (p/i) and included the following parameters: animal survival, neurological deficit and stroke volume. All animal tests were conducted by observers blinded with regard to the treatment groups. All animals were sacrificed at day 30 p/i for histological and PCR analysis. After euthanasia, the animals were decapitated, the brains were removed from the skull and the ischemic portions of the brain tissue were dissected into 3 fragments. One fragment was fixed in 10% formalin for the preparation of the paraffin sections, the other preserved in liquid nitrogen for preparing cryosections and the third preserved in the RNAlater reagent for PCR analysis. Corresponding brain tissue segments of the unaffected contralateral hemisphere were dissected in the same way to be used as a control.

### 4.3. Cell Culture and Conditioned Media Preparation

hiPSCs were derived from human dermal fibroblasts using a CTS CytoTune-iPS 2.1 Sendai Reprogramming Kit (Invitrogen, Carlsbad, CA, USA). Taking a skin biopsy from the donor was approved by the Institutional Ethics Committee of the Research Centre for Medical Genetics (Protocol No. 2019-2/3 from 13 October 2020). To obtain neural stem cells (NSCs) for subsequent differentiation into neuronal and glial lineages, hiPSCs were cultured in DMEM/F12 (Gibco, Waltham, MA, USA) supplemented with N-2 (to 1X, Gibco, Waltham, MA, USA), 2 mM L-glutamine (PanEco, Moscow, Russia), 100 mg/L penicillin-streptomycin (PanEco, Moscow, Russia), 10 µM SB431542 (Stemcell Technologies, Vancouver, BC, Canada), 2 µM dorsomorphin (Stemcell Technologies, Vancouver, BC, Canada) and 200 nM LDN193189 (Sigma-Aldrich, St. Louis, MO, USA) [48]. To obtain NPCs, NSCs were cultured for 2 weeks in DMEM/F12 (PanEco, Moscow, Russia) supplemented with B-27 (to 1X, Gibco, Waltham, MA, USA), 2 mM L-glutamine, 100 mg/L penicillin-streptomycin, 10 ng/mL FGF-2 (ProSpec, Ness-Ziona, Israel) and 1 µM purmorphamine (Stemcell Technologies, Vancouver, BC, Canada) [49]. To obtain GPCs, NSCs were cultured in a DMEM/F12-based growth medium supplemented with N-2, 2 mM L-glutamine, 100 mg/L penicillin-streptomycin and 1% fetal bovine serum (PanEco, Moscow, Russia). The induction process was completed in 3 stages, with each stage being conducted over a period of 3 days. At the first stage, the growth medium was additionally supplemented with 10 ng/mL FGF-2 and 20 ng/mL EGF (ProSpec, Ness-Ziona, Israel). At the second stage, the growth medium was additionally supplemented with 10 ng/mL FGF-2, 20 ng/mL EGF and 20 ng/mL CNTF (PeproTech, Cranbury, NJ, USA). At the third stage, the growth medium was additionally supplemented with 20 ng/mL EGF and 20 ng/mL CNTF [50,51]. To obtain the conditioned media (CM), NPC and GPC cultures from the single donor were washed twice with PBS and incubated in DMEM/F12 (PanEco, Moscow, Russia) for 12 h. The medium was collected from several T175 flasks with cell confluence 80–90% and centrifuged for 5 min at 3000 rpm. The supernatant was passed through a 0.2 µm syringe filter (PanEco, Moscow, Russia) and used in the experiments.

### 4.4. Immunocytochemistry

The cells were fixed in 4% paraformaldehyde solution (PanEco, Moscow, Russia) for 10 min at room temperature; washed with PBS; pre-incubated in 0.25% Triton X-100 and 1% BSA in PBS for 30 min; and incubated with primary antibodies to βIII tubulin (ab7751, Abcam, Cambridge, UK), S100b (ab52642, Abcam, Cambridge, UK), NANOG, SSEA4, OCT4, TRA-1-81, SOX2 (StemLight Pluripotency Antibody Kit, Cell Signaling Technology, Beverly, MA, USA), pancytokeratin (ab7753, Abcam, Cambridge, UK), desmin (ab32362, Abcam, Cambridge, UK), α-fetoprotein (ab3980, Abcam, Cambridge, UK), Nestin (ab105389, Abcam, Cambridge, UK) or PAX6 (ab5790, Abcam, Cambridge, UK) at +4 °C overnight. Secondary antibodies (Alexa Fluor 555 or Alexa Fluor 488 conjugated, Invitrogen, Carlsbad, CA, USA) were applied for 60 min in the dark. The nuclei were counterstained with DAPI (Sigma-Aldrich, St. Louis, MO, USA) solution (1 µg/mL in PBS). The signals were observed, and images were recorded with an Axio Observer.D1 inverted fluorescence microscope equipped with AxioCam HRc camera (Carl Zeiss, Oberkochen, Germany).

### 4.5. Flow Cytometry

The cells were detached with Versene solution (PanEco, Moscow, Russia) and pelleted by centrifugation at 1800 rpm for 5 min, then washed with HBSS (PanEco, Moscow, Russia), fixed in 4% paraformaldehyde for 10 min, washed with PBS, permeabilized with 70% methanol on ice for 10 min, washed twice with PBS and collected by centrifugation at 1800 pm for 5 min. Fixed cells were incubated with primary antibodies to PAX6 (ab5790, Abcam, Cambridge, UK), glial marker S100b (ab52642, Abcam, Cambridge, UK) or neuronal marker βIII tubulin (ab182070, Abcam, Cambridge, UK) at +4 °C for 12 h; washed with PBS; collected by centrifugation at 1800 rpm for 5 min; and incubated with secondary antibodies (Alexa Fluor 488 conjugated, Invitrogen, Carlsbad, CA, USA) for 60 min in the dark. Fixed cells exposed to secondary antibodies were only used as a negative control for the flow cytometry-based quantification. Stained cells were analyzed on a CyFlow ML flow cytometer using the FloMax software (Partec, Goerlitz, Germany). For evaluation of the number of immunopositive cells, the experiment was repeated at least 3 times.

### 4.6. Protein Quantification

To quantify the protein concentration in CM, the Bradford Protein Assay Kit (Bio-rad, Hercules, CA, USA) was used according to the manufacturer’s instructions. Accordingly, 5 μL of each standard or unknown sample was added into a 96 well-plate and then 250 μL of Coomassie reagent was loaded into each well. The plate was then incubated for 5 min at room temperature and the absorbance measured at 595 nm in a plate reader (PerkinElmer, Waltham, MA, USA).

### 4.7. Proteomic Analysis

UltiMate 3000 RSLCnano HPLC system (Thermo Scientific, Waltham, MA, USA) connected to Q-exactive HF mass spectrometer (Thermo Scientific, Waltham, MA, USA) was used for the analysis. Mass spectra were recorded in the ion-positive mode with nanoelectrospray ionization. The identification of proteins was carried out utilizing the SearchGUI v.3.3.16 interface and X!Tandem, OMSSA and MS-GF+ algorithms [52]. The alignments were made with Uniprot database (‘human’-filtered). The structuring of the data by classes and functions of the proteins was carried out using Uniprot and PANTHER databases [53]. For evaluation of the protein composition, the experiment was repeated at least 3 times.

### 4.8. Enzyme-Linked Immunosorbent Assay (ELISA)

The concentrations of GDNF, BDNF, NGF and CNTF in CM were measured by ELISA (RnD systems, Minneapolis, MN, USA) in accordance with the manufacturer’s protocol. The collected CM samples were concentrated 24-fold with 3 kDa Amicon Ultra filter units (Sigma-Aldrich, St. Louis, MO, USA) to 0.5 mg/mL total protein concentrations prior to the analysis. Optical densities (absorbance at 450 nm) were measured in a plate reader (PerkinElmer, Waltham, MA, USA). For evaluation of the concentrations of neurotrophins, the experiment was repeated at least 3 times.

### 4.9. PCR Assay 

For the reverse transcription polymerase chain reaction (RT-PCR) assay, cells or tissues were collected in RNAlater reagent (Thermo Fisher Scientific, Waltham, MA, USA) for the preservation of RNA molecules during storage. The total RNA from the collected cell or tissue samples was isolated with the RNeasy Mini Kit (Qiagen, Limburg, Netherlands) according to the manufacturer’s protocols. cDNA synthesis was carried out with the RevertAid First Strand cDNA Synthesis Kit (Thermo Fisher Scientific, Waltham, MA, USA) according to the manufacturer’s protocol. The real-time PCR mixtures were set up with qPCRmix-HS SYBR (Evrogen, Moscow, Russia); the reactions were carried out in a Bio-Rad iQ thermal cycler as follows: primary denaturation 95 °C for 5 min followed by 40 cycles of denaturation at 95 °C for 20 s, annealing at 55–63 °C for 20 s and elongation at 72 °C for 20 s. Raw data for the genes of interest were normalized against constitutively expressed reference genes *GAPDH* (*Gapdh*, glyceraldehyde-3-phosphate dehydrogenase) and *ACTB* (*Actb*, beta-actin). Expression levels were calculated using the 2^−ΔΔC(T)^ approach. The oligonucleotide sequences are given in Table 2. For evaluation of the mRNA lever of genes in cell cultures, the experiment was repeated at least 3 times. The investigation of the mRNA lever of genes in brain tissue included the animals from the control group (*n* = 10) and the CM recipients (*n* = 12 each).

### 4.10. Modeling of Ischemic Stroke 

Transient 90 min occlusion of the middle cerebral artery in rats was performed by the standard method originally developed by Koizumi [54] and modified by Longa [55], and with MRI guiding as described previously [56]. The animals were anesthetized with a 3.5–4% isoflurane (Aerrane, Baxter HealthCare Corporation, Deerfield, IL, USA) mix with atmospheric air for the induction and 2–2.5% isoflurane/air mix for its maintenance. The right middle cerebral artery was temporary occluded with a silicone rubber-coated monofilament (diameter 0.19 mm, length 30 mm, diameter with coating 0.37 ± 0.02 mm, coating length 3–4 mm; Doccol, Sharon, MA, USA) for 90 min. During the operation and until the emergence from anesthesia, the body temperature was maintained at 37 °C with a warming pad.

### 4.11. Intra-Arterial Administration

Intra-arterial infusion was performed 24 h after MCAO for all experimental groups. Under isoflurane inhalation anesthesia, as described above, the right common carotid artery (CCA), the external carotid artery (ECA) stump and the internal carotid artery (ICA) were exposed, and the pterygopalatine artery was ligated by a 5 ± 0 silk suture. A microcatheter (rodent tail vein catheter with diameter 1F, Braintree Scientific, Inc., Braintree, MA, USA) filled with saline to prevent air bubbles was inserted into the stump of the ECA and advanced into the ICA for 5–6 mm from the bifurcation of the CCA. The catheter external diameter was small enough to allow blood flow around it during the infusion. The catheter was then connected to a 1 mL syringe placed in the microinjector, and 1 mL of concentrated GPC-CM (50 µg/mL of total protein) or NPC-CM (50 µg/mL of total protein), or 1 mL of non-conditioned DMEM/F12-based medium for the control group was delivered into the ICA with the infusion velocity 100 μL/1 min with the maintenance of blood flow in ICA. After intra-arterial administration, the catheter was removed, the ECA stump was electrocoagulated and the incision was closed with a 5 ± 0 silk suture.

### 4.12. Assessment of the Neurological Deficit and Functional Recovery 

Therapeutic effects of CM administration were estimated immediately before the infusion (day 1) and on days 7, 14 and 30 p/i by the assessment of the survival rates, infarct volume evaluated by MRI and neurological deficits evaluated by the modified neurological severity score (mNSS) for rodents [23].

### 4.13. Magnetic Resonance Imaging (MRI) 

The imaging was accomplished in a 7T ClinScan small animal MRI system (Bruker BioSpin, Billerica, MA, USA). During the procedure, rats were maintained under isoflurane inhalation anesthesia as described above. For MRI evaluation of the in vivo dynamics of the stroke volume, axial plane T2-weighted brain images (T2-WI) were acquired immediately before the infusion (day 0) and on days 7, 14 and 30 p/i. Quantitative reconstruction of the infarct volume on the basis of T2-WI was accomplished by the ImageJ software (Wayne Rasband, National Institute of Mental Health, Bethesda, MD, USA) by calculating V= (S_1_ + … + S_n_) × (h + d), where S values correspond to the infarct area measured in individual sections, h is slice thickness, and d is the spacing between the slices.

### 4.14. Histology and Immunohistochemistry

The formalin-fixed tissues were dehydrated and embedded in paraffin and cut on a microtome. The 5–7 µm thick sections were positioned on gelatin-coated slides and dried at 37 °C for 24 h. The sections were deparaffinized in xylene and rehydrated in 100–70° ethanol series, stained with H&E, dehydrated and mounted.

For immunohistochemistry, the tissues were cryosectioned at a 4–5 µm thickness. The sections were pre-blocked in PBS with 0.3% Triton X-100 and 2% BSA for 1 h; incubated with primary antibodies anti-CD68 (ab125212, Abcam, Cambridge, UK), anti-CD86 (ab213044, Abcam, Cambridge, UK), anti-CD206 (ab64693, Abcam, Cambridge, UK), anti-CD34 (ab185732, Abcam, Cambridge, UK) and anti-CD11b (sc-53086, Santa Cruz Biotechnology, Dallas, TX, USA) at +4 °C overnight; washed and incubated with secondary antibodies (Alexa Fluor 488 conjugated, Invitrogen, and Alexa Fluor 555 conjugated, Invitrogen, Carlsbad, CA, USA) for 60 min in the dark. The nuclei were counterstained with DAPI solution (1 µg/mL in PBS). The images were recorded with Axio Observer.D1 inverted fluorescence microscope equipped with an AxioCam HRc camera (Carl Zeiss, Oberkochen, Germany). Areas in and around the site of injury were randomly selected from 7–10 locations for each animal (control group *n* = 10 and CM recipients *n* = 12 for each group) to obtain positively stained cells or blood vessels. The total counts of CD68+, CD11b+, CD86+ and CD206+ cells and the formation of blood vessels (anti-CD34 stained histological sections) were determined with ImageJ software by counting the positively stained cells or blood vessels at x200 magnification on days 30 p/i, which were then normalized to per sq mm of the section.

### 4.15. TUNEL Assay

The TUNEL assay was carried out on cryosections according to the manufacturer’s instructions (Click-iT TUNEL Alexa Fluor Imaging Assay, Invitrogen, Carlsbad, CA, USA). Finally, all sections were washed with PBS and nuclei were counterstained with DAPI (Sigma-Aldrich, St. Louis, MO, USA) solution (1 µg/mL in PBS). The sections were observed with Axio Observer.D1 inverted fluorescence microscope equipped with AxioCam HRc camera (Carl Zeiss, Oberkochen, Germany). TUNEL-positive cells were counted using ImageJ analysis software at x400 magnification on days 30 p/i. Areas in and around the damage tissue were randomly selected from 7–10 locations for each animal (control group *n* = 10 and CM recipients *n* = 12 for each group) to obtain counts of positively stained cells, which were then normalized to per sq mm of the section.

### 4.16. Statistical Analysis 

The data were processed in SigmaPlot 12.5 software. Results were expressed as the mean with standard deviation (SD). Differences at *p* ≤ 0.05 were considered significant. Animal survival was assessed by the Kaplan–Meier method using a log-rank test. For estimation of mNSS, infarct volume, PCR and morphometric analysis data were carried out by t-test in the case of normal distribution or Mann–Whitney test in the case of non-normal distribution for pairwise comparisons between groups. For multiple comparisons, one-way ANOVA with the Holm–Sidak method was used for the cases of normal distribution, whereas ANOVA on ranks with Dunn’s test was used for the cases of non-normal distribution. The values of mNSS score and infarct volume were normalized based on the data captured on day 1 for better assessment of the dynamics of changes.

## Figures and Tables

**Table 1:** Secreted proteins of the hiPSC-derived neuronal and glial progenitor cells identified by a proteomic approach.

Biological Processes	Neuronal Progenitor Cells (NPCs)	Glial Progenitor Cells (GPCs)
regulation of apoptosis and cell survival	tissue inhibitor of metalloproteinases 2 (TIMP2)	heat shock 70 kDa protein 4 (HSPA4)
	secretogranin-2 (chromogranin C, SCG2)	heat shock protein 105 kDa (HSPH1)
	Wnt family member 5a (WNT5A)	Hsc70-interacting protein (ST13)
	neuropilin-1 (NRP1)	leukemia inhibitory factor (LIF)
	Ras homolog family member A (RHOA)	growth arrest-specific protein 6 (GAS6)
	platelet-derived growth factor D (PDGFD)	gremlin 1 (GREM1)
		tetranectin (TETN)
influence on immune cells	annexin A1 (ANXA1)	collectin subfamily member 12 (COLEC12)
	nectin 2 (NECTIN2)	vitamin D binding protein (VTDB)
	meteorin-like protein (METRNL)	importin subunit beta 1 (KPNB1)
	moesin (MSN)	Toll-interacting protein (TOLLIP)
	apolipoprotein A1 (APOA1)	S100-A11 protein (S100A11)
	osteopontin (SPP1)	growth and differentiation factor 15 (GDF15)
	28 kDa heat- and acid-stable phosphoprotein (PDAP1)	SH3 domain-binding glutamic acid-rich-like protein 3 (SH3BGRL3)
	Ras-related protein Rap-1b (RAP1B)	
angiogenesis	lactadherin (MFGE8)	myeloid-derived growth factor (MYDGF)
	secretogranin 3 (SCG3)	transforming growth factor-β2 (TGFB2)
	glypican-1 (GPC1)	

**Table 2:** Sequences and annealing temperatures of PCR primers.

Target	Sequence (5′ to 3′)	Annealing Temperature, °C
*Bax*	for TTGTGGCTGGAGTCCTCACT rev TTTCCCCGTTCCCCATTCATC	63
*Bcl2*	for GGGGCTACGAGTGGGATACT rev GACGGTAGCGACGAGAGAAG	62.5
*Tnf*	for CCACCACGCTCTTCTGTCTA rev GCTACGGGCTTGTCACTCG	60.1
*Il4*	for ATGTAACGACAGCCCTCTGA rev AGCACGGAGGTACATCACG	56.6
*Il10*	for GCCCAGAAATCAAGGAGCAT rev TGAGTGTCACGTAGGCTTCTA	58.5
*Il13*	for CCAGAAGACTTCCCTGTGCA rev CCCTCAGTGGCCATAGCG	62.3
*Gapdh*	for GAGATTACTGCCCTGGCTCC rev GCTCAGTAACAGTCCGCCTA	56.6
*Actb*	for GCGAGATCCCGCTAACATCA rev CCCTTCCACGATGCCAAAGT	55
*GDNF*	for GAAAAGGTCGGAGAGGCCAG rev AGCCGCTGCAGTACCTAAAA	63
*BDNF*	for CCAGGTGAGAAGAGTGATGACC rev CCTTGTCCTCGGATGTTTGC	62.6
*CNTF*	for GTAAACCCAGCTGACTTGTTTCC rev CAGCGGTGAATGCTCTGTGA	55.5
*NGF*	for ACCCGCAACATTACTGTGGACC rev GACCTCGAAGTCCAGATCCTGA	55
*MAP2*	for CTCAGCACCGCTAACAGAGG rev CATTGGCGCTTCGGACAAG	62
*TUBB3*	for GCCAGACGCGCCCAGTATGAGG rev GGTTCCGGGTTCCAGGTCCAC	62
*S100B*	for AGCTTTCCAGCCGTGTTGTA rev ACGGTGCACGCTTTATCACT	60.1
*ENO2*	forAGCCTCTACGGGCATCTATGA rev TTCTCAGTCCCATCCAACTCC	62
*GFAP*	for CTGCGGCTCGATCAACTCA rev TCCAGCGACTCAATCTTCCTC	61
*GREM1*	for GTGACGGAGCGCAAATACCT rev GGTTGATGATGGTGCGACTG	56.7
*GAS6*	for ATCCTCCTCTTTGCCGGAGG rev CGACACCGTTGTAGCGCAG	59.1
*GDF15*	for CACCTGCGTATCTCTCGGGC rev TCACGTCCCACGACCTTGAC	59.7
*LIF*	for GAGCTGTACCGCATAGTCGT rev CACAGCACGTTGCTAAGGAG	56.7
*MYDGF*	for CCACGACGGTGGCGTTTG rev CCCGGGCCCACGTTATGG	60
*TGFb2*	for ACATTGGCAAAACACCCAAGA rev GTGTCTGAACTAGTACCGCCTTT	55.6
*TWF2*	for TAGAGCGGGAAACCATTGAG rev TCATGGGTGTGCTTGTAGAG	54.5
*SNX3*	for GGCAGCAGCTACAGCGAAATG rev GTCATTCAGGTTCTGCGGCTT	58.0
*NPTX2*	for TTGGACAAGAGCAGGACACC rev GAGCAGTTGGCGATGTTGAC	57.0
*FGF8*	for GCTGCAGAATGCCAAGTACGA rev TCATGAAGTGGACCTCACGCT	58.1
*EFNB1*	for GTTGGGCAAGATCCCAATGC rev GGCCACTCTTCTCTTCCTGG	57.0
*NTN1*	for CTTCCTCACCGACCTCAACAA rev CGAACTTCTTGCCGAGGGAC	57.6
*VGF*	for TGAGCATAAAGAGCCGGTAGC rev GGAAAAGCTCTCCCTCGTCCT	58.1
*SERPINI1*	for TGGTAACTGCTAAAGAGAGCCA rev TGGCCACGGCTACATTTTGA	56.5
*GAPDH*	for GAAGGTGAAGGTCGGAGTCA rev TTCACACCCATGACGAACAT	56.6
*ACTB*	for CCTGGCACCCAGCACAAT rev GGGCCGGACTCGTCATAC	58.2

R. norvegicus and H. sapiens gene-specific oligonucleotides.

## Data Availability

The data presented in this study are openly available on the pre-print server Research Square (https://www.researchsquare.com/ (accessed on 28 April 2021)) and the URL is https://www.researchsquare.com/article/rs-49227/v2 (accessed on 28 April 2021) at DOI: 10.21203/rs.3.rs-49227/v2, 28 April 2021.

## Supplementary Materials

The following are available online at https://www.mdpi.com/article/10.3390/ijms22094694/s1, Table S1. Proteomic analysis of the GPC-CM and NPC-CM (all data).
